# Statin and Cancer Mortality and Survival: An Umbrella Systematic Review and Meta-Analysis

**DOI:** 10.3390/jcm9020326

**Published:** 2020-01-23

**Authors:** Gwang Hun Jeong, Keum Hwa Lee, Jong Yeob Kim, Michael Eisenhut, Andreas Kronbichler, Hans J. van der Vliet, Jae Il Shin, Gabriele Gamerith

**Affiliations:** 1 College of Medicine, Gyeongsang National University, Jinju 52727, Korea; gwangh.jeong@gmail.com; 2 Department of Pediatrics, Yonsei University College of Medicine, Yonsei-ro 50, Seodaemun-gu, C.P.O. Box 8044, Seoul 03722, Korea; AZSAGM@yuhs.ac; 3 Yonsei University College of Medicine, Seoul 03722, Korea; crossing96@yonsei.ac.kr; 4 Luton & Dunstable University Hospital NHS Foundation Trust, Lewsey Road, Luton LU4 ODZ, UK; michael_eisenhut@yahoo.com; 5 Department of Internal Medicine IV (Nephrology and Hypertension), Medical University Innsbruck, 6020 Innsbruck, Austria; andreas.kronbichler@i-med.ac.at; 6 Department of Medical Oncology, Amsterdam UMC, Cancer Center Amsterdam, VU University, 1081 HV Amsterdam, The Netherlands; jj.vandervliet@amsterdamumc.nl; 7 Internal Medicine V, Department of Hematology and Oncology, Medical University Innsbruck, 6020 Innsbruck, Austria

**Keywords:** statin, cancer mortality, cancer survival, meta-analysis, umbrella review

## Abstract

The aim of this study is to provide an overview and understand the strength of evidence and the extent of potential biases and the validity of claimed associations between the use of statins and cancer mortality or survival. We performed a comprehensive umbrella review of meta-analyses and systematically appraised the relevant meta-analyses of observational studies on the associations between statin use and cancer mortality or survival in various kinds of cancer. We searched the PubMed database and screened the reference list of relevant articles. We obtained the summary effect, 95% confidence interval, heterogeneity, and also examined small study effects and 95% prediction intervals for effect sizes, and the level of evidence was determined from the criteria. Regarding cancer mortality, statin use showed convincing evidence for an association with a reduced cancer-specific mortality rate for colorectal cancer. Four associations with reduced all-cause mortality (for breast cancer, colorectal cancer, endocrine-related gynecological cancer, and ovarian cancer) had a suggestive evidence. Moreover, analyses in nine cancers showed a weak level of evidence, while the remaining 15 did not indicate significant changes in either direction. Although there was a preventive effect of statin on cancer mortality in some cancer types, the evidence supporting the use of statins to reduce cancer mortality or survival was low.

## 1. Introduction

Cancer is a disease caused by an uncontrolled division of abnormal cells in a part of the body due to breakdown in the processes which control cell proliferation, differentiation, and death of particular cells. According to the recent incidence and mortality data, there were an estimated 14.1 million new cancer cases and 8.2 million cancer deaths worldwide in 2012 [1]. Recently, the relative survival of patients with cancer has gradually increased over time [2] and the new anti-cancer regimens have broadened the therapeutic options for many cancer patients significantly. Nevertheless, cancer mortality is still high in most cancer types.

β-Hydroxy β-methylglutaryl-CoA (HMG-CoA), also known as 3-hydroxy-3-methylglutaryl-CoA, is an intermediate in the mevalonate and ketogenesis pathways, and its inhibitors (statins) have been used to reduce plasma cholesterol levels for preventing coronary heart disease [3]. Although there have been earlier concerns on the carcinogenicity of statins, such as initiation or promotion of cancers in rodents at concentrations equivalent to those commonly prescribed in humans [4], there has also been growing evidence which suggests that statins could have a chemopreventive effect against cancer [5,6]

Although the potential mechanisms of the anti-cancer effect of statin are still elusive, inhibition of cancer cell growth, promotion of apoptotic cell death and inhibition of matrix metalloproteinases are involved in processes such as tumor growth, invasion, and metastasis have been suggested [7,8,9].

Recently, there have been many epidemiologic studies and meta-analyses on the beneficial effect of statin on cancer incidence and mortality or survival. However, the results were conflicting on these associations [10,11,12]. In addition, some of these reported associations could be caused by biases. We have previously examined the association between statin use and cancer incidence by performing an umbrella review by assessing the level of evidence [13], but there has been no study focusing on cancer mortality. Therefore, to provide an overview of the strength of evidence, the extent of potential biases and the validity of the claimed associations between statin use and cancer mortality or survival, we performed an umbrella review of the evidence across the published meta-analyses on the associations between statin use and various kinds of cancer mortalities or survivals.

## 2. Methods

We performed an umbrella review of meta-analyses and systematic reviews on the associations between statin use and cancer mortality or survival. This umbrella review and meta-analysis was conducted according to the Preferred Reporting Items for Systematic Reviews and Meta-Analyses (PRISMA) guidelines [14]. The PRISMA checklist is shown in Supplementary Materials.

### 2.1. Methods for Literature Search

**Figure 1:**
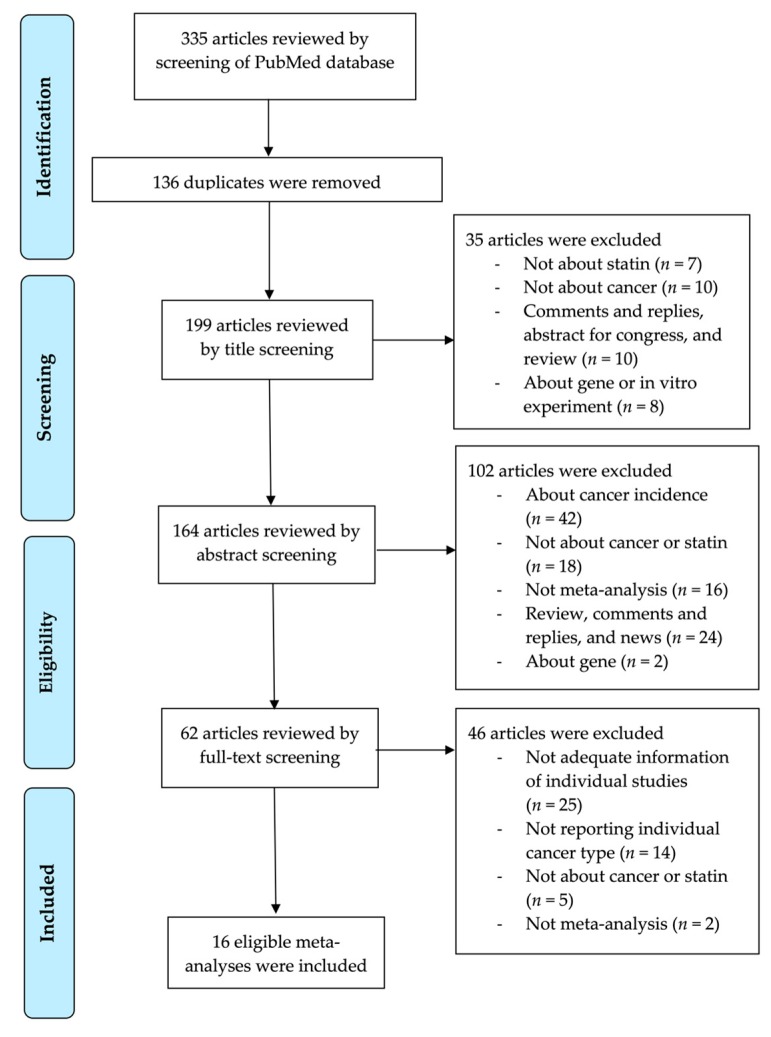
Flow diagram of literature search.

Two investigators (G.H.J. and J.I.S) searched the data and any discrepancies were resolved by discussion and consensus. We searched the literature using the PubMed database and selected the articles written in English. The last search was performed in August 2018. The following keywords were used to find the relevant articles: ‘(hydroxymethyl glutaryl-CoA reductase inhibitor OR statin) AND (cancer OR neoplasm OR tumor OR malignancy) AND (meta-analysis OR systematic review) AND (survival OR mortality OR death)’. We carefully reviewed the retrieved articles by examining titles, abstracts and full texts, and then determined whether the article could be included or excluded. The detailed search strategy is shown in Figure 1.

### 2.2. Eligibility Criteria and Data Extraction

We included meta-analyses and systematic reviews of both RCTs and observational studies (cohort or case-control studies) on the relationship between statin use and cancer mortality or survival. However, we could not find any meta-analysis of RCTs on this association and therefore, only observational studies were included and analyzed. Any mortality or survival outcomes were included and five outcomes (all-cause mortality, cancer-specific mortality, recurrence-free survival, progression-free survival, and disease-free survival) were found in the included meta-analyses on the associations between statin use and cancer mortality or survival. Narrative review articles, in vitro or animal studies and genetic association meta-analyses were excluded in our study. Meta-analyses with insufficient data were also excluded. If there are several meta-analysis articles on the same topic and outcome, we included all the meta-analyses to see whether there are any discrepancies among them.

We obtained the original data from eligible meta-analyses, and extracted and summarized information on the first author, year of publication, the type of cancer, the type of outcome (all-cause mortality, cancer-specific mortality, recurrence-free survival, progression-free survival, and disease-free survival), the study design, the number of included studies, the number of case and total participants, and the random effects with 95% CI. In addition, we extracted the raw datasets of each individual study for the further re-analysis of meta-analysis.

### 2.3. Statistical Analysis

We firstly reanalyzed each meta-analysis result for the relationship between statin use and cancer mortality or survival. We found that most published meta-analyses presented only the results of random effects and therefore, we performed not only random-effects meta-analysis but also fixed-effect meta-analysis. In addition, if there were overlapping meta-analyses on the same topic, we pooled all the datasets of individual studies from eligible meta-analyses according to the type of cancer or study design and performed re-meta-analysis after eliminating the overlapping individual studies and including missing individual studies. We presented the summary effect size, 95% CI and *p*-value with both random- and fixed-effects. All re-analyses in this study were performed using Comprehensive Meta-Analysis software ver.3.3.070 (Borenstein, Englewood, NJ, USA).

For each meta-analysis, we re-analyzed the individual studies and estimated the summary effects and 95% CI using both fixed and inverse variance random- and fixed-effects methods [15]. We also calculated and presented the 95% prediction intervals (PIs), which address the dispersion of effects in 95% of cases the true effect in a new study will fall within the PIs and further account for between-study heterogeneity [16], whereas CI reflect the accuracy of the mean.

Heterogeneity across the individual studies was assessed using I^2^ metric of inconsistency and the *p*-value of the Cochrane Q test [17]. Publication bias was evaluated by using Egger’s regression test [18]. Small study effects were used to detect publication and report bias [19,20]. If Egger’s regression test was significant (*p*-value < 0.10) in random-effects meta-analyses, we judged that the meta-analysis has small-study effects.

### 2.4. The Criteria to Determine the Level of Evidence

We determined the level of evidence for each reanalyzed meta-analysis or pooled meta-analysis to strengthen the associations between statin use and cancer mortality or survival. The criteria to determine the level of evidence were classified according to the statistical significance by random and fixed-effects *p*-value, 95% PI, a small-study effect, a between-study heterogeneity and concordance between the result of the largest study among each meta-analysis and that of meta-analysis [13,21]. The level of evidence for the association was determined as follows:

#### 2.4.1. Convincing Evidence

There was a strong statistical significance in fixed-effects and random-effects meta-analyses at *p*-value < 0.001, 95% PI excluded null, there was no large between-study heterogeneity and no small study effects. There was a concordance between the result of the largest study and that of meta-analysis

#### 2.4.2. Suggestive Evidence

The significance threshold was crossed for the random summary effects (*p* < 0.05), but 95% PI included the null and there was not large between-study heterogeneity and there were no small study effects.

#### 2.4.3. Weak (Probable) Evidence

The significance threshold was crossed for the random summary effects (*p* < 0.05), but 95% PI included the null, there was large between-study heterogeneity or small study effects.

#### 2.4.4. Nonsignificant Associations

The significance threshold was not crossed for the random summary effects (*p* > 0.05). However, if the heterogeneity was large, we rechecked the results whether it may be due to differences in the direction of the effect or it can be due to differences in the size of the association although all studies may show increased risk. In the latter case, we re-determined the level of evidence again [13,21].

## 3. Results

### 3.1. Search Strategy for the Literature and Included Studies for Reanalysis

A total of 335 meta-analyses were retrieved from our PubMed database search. 136 duplicate articles were initially excluded, and an additional 35 articles were screened by title. Another 102 articles were excluded after assessing the abstract, and 46 articles were finally excluded after full-text screening and finally, 16 eligible meta-analyses reporting various kinds of cancer mortality or survival in 11 cancers were finally selected for re-analysis (Figure 1) [22,23,24,25,26,27,28,29,30,31,32,33,34,35,36,37]. Overall, all-cause mortality was reported as outcomes in 11 cancer types, cancer-specific mortality in 8 cancer types, recurrence-free survival in 5 cancer types, progression-free survival in 4 cancer types and disease-free survival in one cancer type (Table 1, Table 2, Table 3 and Table 4).

### 3.2. The Effect of Statin on All-Cause Mortality in 11 Cancers

The results of each meta-analysis on the effect of statin on all-cause mortality in various cancer types are summarized in Table 1 and the results of meta-analyses in which all the individual datasets are pooled are summarized in Table 4.

There were no associations between statin use and all-cause mortality in three cancer types (bladder, endometrial and urothelial tract cancer). In breast cancer, three meta-analyses which included any statin use (all weak evidence due to high heterogeneity) and one with pre-diagnostic statin use (suggestive evidence) showed the beneficial effect of statin on all-cause mortality, while there was no significant association between post-diagnostic statin use and all-cause mortality in one meta-analysis. When the individual datasets were all pooled (*n* = 20), the evidence for the effect of statin use in preventing all-cause mortality in breast cancer was suggestive despite a high heterogeneity because it was due to differences in the effect size of the association.

In colorectal cancer, nine of the 10 meta-analyses showed the beneficial effect of statin on all-cause mortality, while only one older meta-analysis showed no significant association between post-diagnostic statin use and all-cause mortality. When the individual datasets were all pooled (*n* = 24), the evidence for the effect of statin use in preventing all-cause mortality in colorectal cancer was suggestive despite a high heterogeneity, because it was due to differences in the effect size of the association.

In endocrine-related gynecological cancer, there was only one meta-analysis (*n* = 9) which showed a beneficial effect of statin on all-cause mortality with suggestive evidence.

In kidney cancer, there were two meta-analyses that showed a beneficial effect of statin on all-cause mortality with one suggestive and the other weak evidence. When the individual datasets were all pooled (*n* = 7), the evidence for the effect of statin use in preventing all-cause mortality in kidney cancer was weak due to small study effects and high heterogeneity.

In ovarian cancer, there were three meta-analyses that showed a beneficial effect of statin on all-cause mortality with one convincing, the other suggestive and another not estimable. When the individual datasets were all pooled (*n* = 7), the evidence for the effect of statin use in preventing all-cause mortality in ovarian cancer was suggestive.

In pancreatic cancer, there was only one meta-analysis (*n* = 6) which showed a beneficial effect of statin on all-cause mortality with weak evidence due to small study effects and high heterogeneity.

In prostate cancer, five of the six meta-analyses showed the beneficial effect of statin on all-cause mortality (two suggestive, two weak and one not estimable), while only one older meta-analysis showed no significant association between post-diagnostic statin use and all-cause mortality. When the individual datasets were all pooled (*n* = 21), the evidence for the effect of statin use in preventing all-cause mortality in prostate cancer was weak due to small study effects and high heterogeneity.

In urothelial tract cancer, there was only one meta-analysis (*n* = 5) which showed a beneficial effect of statin on all-cause mortality with weak evidence due to high heterogeneity.

### 3.3. The Effect of Statin on Cancer-Specific Mortality in 8 Cancers

The results of each meta-analysis on the effect of statin on cancer-specific mortality in various cancer types are summarized in Table 2 and the results of meta-analyses in which all the individual datasets are pooled are summarized in Table 4.

There was only one meta-analysis for each in three cancer types (bladder, endocrine-related gynecological cancer, and urothelial tract cancer) and no associations were found between statin use and cancer-specific mortality in each cancer type.

In breast cancer, four of the five meta-analyses showed the beneficial effect of statin on cancer-specific mortality (one suggestive, two weak and one not estimable, but at least suggestive), while only one meta-analysis showed no significant association. When the individual datasets were all pooled (*n* = 28), the evidence for the effect of statin use in preventing cancer-specific mortality in breast cancer was weak due to small study effects and high heterogeneity.

In colorectal cancer, eight of the nine meta-analyses showed the beneficial effect of statin on cancer-specific mortality, while only one older meta-analysis showed no significant association between post-diagnostic statin use and cancer-specific mortality. When the individual datasets were all pooled (*n* = 13), the evidence for the effect of statin use in preventing cancer-specific mortality in colorectal cancer was convincing.

In kidney cancer, there were two meta-analyses that showed a beneficial effect of statin on cancer-specific mortality with one weak and the other not significant in older meta-analysis. When the individual datasets were all pooled (*n* = 6), the evidence for the effect of statin use in preventing cancer-specific mortality in kidney cancer was weak due to high heterogeneity.

In ovarian cancer, there was only one meta-analysis (*n* = 3) which showed a beneficial effect of statin on cancer-specific mortality with weak evidence due to the presence of small study effect.

In prostate cancer, all six of the meta-analyses showed the beneficial effect of statin on cancer-specific mortality (five suggestive and one weak). When the individual datasets were all pooled (*n* = 15), the evidence for the effect of statin use in preventing cancer-specific mortality in prostate cancer was weak due to small study effects and high heterogeneity.

### 3.4. The Effect of Statin on Recurrence-Free Survival, Progression-Free Survival, and Disease-Free Survival

The results of each meta-analysis on the effect of statin on recurrence-free survival, progression-free survival or disease-free survival in various cancer types are summarized in Table 3 and the pooled results of meta-analyses in recurrence-free survival for kidney cancer are summarized in Table 4.

Recurrence-free survival was measured as outcome for the preventive effect of statin in five cancers (bladder, breast, colorectal, kidney and prostate cancer) and no associations were found between statin use and recurrence-free survival in each cancer type in four cancers (bladder, colorectal, kidney and prostate cancer) and there was only one meta-analysis (*n* = 10) which showed a beneficial effect of statin on recurrence-free survival in breast cancer with a suggestive evidence.

Progression-free survival was measured as an outcome for the preventive effect of statin in four cancers (bladder, endocrine-related gynecological cancer, kidney, and prostate cancer) and no associations were found in these cancers.

Disease-free survival was measured as an outcome for the preventive effect of statin in one cancer (colorectal cancer) and no association was found.

## 4. Discussion

The purpose of this umbrella review of previous meta-analysis and re-analysis of meta-analyses, including all the individual studies was to highlight the potential effects of statin use on cancer mortality or survival. Our team recently examined the association between statin use and cancer incidence, and we found out that there were substantial weak or not significant associations [13]. We further analyzed the data from 16 meta-analyses to evaluate the use of statins and cancer mortality or survival. With only using random-effects *p*-value and effect size with 95% confidence interval (CI) [38], which is a conventional interpretation of current meta-analysis, 14 of 29 associations of cancer mortality or survival showed a statistically significant preventive effect of statin on these outcomes. Among these outcomes, the use of statin significantly decreased the cancer-specific mortality of colorectal cancer supported with a convincing level of evidence. These studies of colorectal cancer on cancer-specific mortality were performed with four meta-analyses with 13 individual studies including a total of 118,996 patients. The main findings with the determined level of evidence were summarized in Table 5.

This outcome can be fortified with the preclinical studies of the anticancer effects of adjuvant statin on colorectal cancer [39]. Several mechanisms responsible for anticancer effect on colorectal cancer were inducing apoptosis by down-regulating of anti-apoptotic proteins [40], inhibition of cellular proliferation [41], or inhibition of angiogenesis [42]. Recent studies present that statin inhibits the formation of mevalonate from HMG-CoA, it subsequently inhibits the Ras/Rho prenylation and downstream reactions, expected to overcome the resistance of anti-epidermal growth factor receptor (EGFR) therapy in patients with K-RAS mutation [43]. These beneficial effects of statin on cancer, especially on colorectal cancer, may largely overlapping and can be applied both in the field of cancer prevention and adjuvant cancer therapy.

However, besides the case of colorectal cancer, statin’s effect on other types of cancer mortality was supported by a weak or non-significant level of evidence. This can be addressed with possible explanations. First, there was a relatively limited number of studies. Colorectal and breast cancer show a high rate of incidence and mortality worldwide, which leads to a large number of RCTs and observational studies [44]. However, underpowered-studies supported by the small number of studies have limitations to be validated [45]. Due to this reason, further updated meta-analyses should include a large number of individual studies. Also, some cancers innately have poor prognosis or can be detected in a more progressed stage. Cancers such as bladder or pancreatic cancer have a relatively low survival rate in the general population, which may attenuate the effect of statin’s benefit.

Among pooled meta-analyses of all-cause mortality of patients with 11 types of cancer, eight of them showed a negative association and four of them had a suggestive level of evidence (Table 4). Above all, statin showed a significantly conspicuous effect on all-cause mortality of breast and colorectal cancer patients, supported by a sufficient number of meta-analyses with suggestive or convincing level. Besides, in case of breast cancer, the disease-specific mortality (in this case, breast cancer-specific mortality) showed a negative association with the use of statin, supported by the lower magnitude of effect size and weaker level of evidence. All-cause mortality is a widely adopted result variable of many trials and observational studies because it can present any unexpected lethal outcomes of trials, such as non-cardiovascular outcomes in patients with hyperlipidemia [46]. However, this should be carefully interpreted since most of the statin users have risk factors of cardiovascular diseases or higher rate of cardiovascular death, use of statin can act as confounding factors of the outcome measures.

Most of the results observed the survival rate of cancer (recurrence-free survival, progression-free survival, and disease-free survival) were shown to have non-significant results, except for the results of breast cancer recurrence-free survival. Since studies of these kinds of outcome measures have not widely performed, the sample sizes of individual meta-analyses are limited, which may lead to false-positive estimates. In case of breast cancer recurrence-free survival, it only includes one meta-analysis [25], but with an adequate number of individual studies and sample sizes. To robust the statistical significance, further study should be conducted.

Meta-analysis is an important research design for appraising evidence and guiding medical practice and health policy by combining data from many studies and umbrella review (reviews of previously published systematic reviews or meta-analyses) emerged as an important method of evidence synthesis because it can provide a wider picture compared with a meta-analysis which is limited to one treatment comparison or even one outcome [47]. Recently, however, mass production of flawed meta-analysis also has been a problem in the medical field [48]. Meta-analysis has its own several limitations such as heterogeneity or publication bias. Also, overlapping meta-analyses on the same topic have been an important issue [49,50], because they often show conflicting results among them and our umbrella review also showed that there were many overlapped meta-analyses with discordant results. The most updated meta-analysis should include all the previous individual studies, but some previous studies are frequently missing in the last meta-analysis despite extensive search strategy due to not reviewing the previous meta-analysis on the same topic, which can lead to misleading results. There are several ways to overcome this problem, and therefore, we performed re-analysis by pooling all the individual studies datasets in addition to analysis of each meta-analysis.

Due to these several problems, the results of meta-analysis should be interpreted with caution and recent umbrella reviews suggest the use of several criteria for determining level of evidence such as the degree of *p*-value, the statistical significance in both random and fixed effect models, between-study heterogeneity, small study effect, and 95% PI, which is more strict than the *p*-value alone [20,21,51]. The strength of evidence reinforces the results from the meta-analyses and assists to choose the best evidence for the decision.

Our study has several limitations: (1) we only included the re-analyzable meta-analyses for re-analysis, (2) potential confounding factors differed across the individual studies (3) individual observational studies themselves can have biases, (4) each meta-analysis might include erroneous individual studies, (5) some statistics such as 95% PI and Egger’s tests cannot be done if there were small number of individual studies, (6) the criteria we used may not be definitive criteria for assessing the strength of evidence, (7) the dose-effect of statin was beyond the scope of our analysis, (8) and the subgroup analyses of the effects such as adjuvant therapy or underlying patients’ conditions were also beyond our scope and were not performed due to lack of studies for the analysis. Future studies should be performed considering limitations of individual meta-analyses and potential biases, and also should consider the dose-dependent effect of statin.

## 5. Conclusions

Our umbrella review extensively re-analyzed the meta-analyses on the associations between statin use and cancer mortality or survival. 14 of 29 studies on statin-cancer mortality were significant. Especially, the use of statin was significantly associated with a reduction of cancer-specific mortality of colorectal cancer supported by a convincing level of evidence, which can be interpreted that it has a noteworthy association. Although there have been extensive epidemiologic or meta-analysis studies on the associations of statin use with cancer mortality or survival and report many strong claims of significance for the associations, only a minor portion of these associations have convincing or suggestive associations without biases. Our findings would give a clue to clinicians and researchers and help understand the true associations.

## Figures and Tables

**Table 1:** Summary of each individual meta-analysis on associations of the use of statin and all-cause mortality in various cancers.

Type/Author, Year	Study Design	No of Study	No of Total Participants	Random Effects (Reported) (ES, 95%CI)	Random Effects (Re-Analyzed) (ES, 95%CI)	Fixed Effects (Re-Analyzed) (ES, 95%CI)	Largest Effect §	Egger	I^2^ (*P*) †	*P* (Random)	*P* (Fixed)	95% PI (Random)	Small Study Effect	Same Direction	Evidence
Bladder cancer															
Luo 2015	Obs	1	1117	1.14 (0.89–1.44)	1.14 (0.89–1.44)	1.14 (0.89–1.44)	1.14 (0.89–1.44)	-	-	0.286	0.286	NA	-	No	Non-significant
Breast cancer															
Mei 2017	Cohort	7	24,255	0.65 (0.43–0.99)	0.65 (0.43–0.98)	0.72 (0.67–0.77)	0.78 (0.72–0.84)	0.541	92.7 (<0.001)	0.042	<0.001	0.15–2.76	No	Yes	Weak
Liu 2017	Cohort	8	68,373	0.72 (0.58–0.89)	0.72 (0.58–0.89)	0.74 (0.69–0.78)	0.46 (0.38–0.55)	0.702	87.4 (<0.001)	0.002	<0.001	0.36–1.44	No	Yes	Weak
Manthravadi 2016	Cohort	8	40,756	0.66 (0.44–0.99)	0.66 (0.44–0.99)	0.64 (0.57–0.70)	0.39 (0.33–0.46)	0.864	89.0 (<0.001)	0.043	<0.001	0.18–2.40	No	Yes	Weak
Zhong 2015 (post-diagnostic)	Obs	6	51,265	0.75 (0.55–1.02)	0.75 (0.55–1.03)	0.72 (0.64–0.80)	0.47 (0.38–0.59)	0.816	77.8 (<0.001)	0.079	<0.001	0.27–2.09	No	No	Non-significant
Zhong 2015 (pre-diagnostic)	Obs	3	49,116	0.73 (0.62–0.86)	0.73 (0.63–0.86)	0.76 (0.69–0.83)	0.60 (0.45–0.81)	0.501	33.3 (0.269)	<0.001	<0.001	0.18–2.95	No	Yes	Suggestive
Colorectal cancer															
Mei 2017	Cohort	9	44,476	0.76 (0.68–0.86)	0.76 (0.68–0.86)	0.75 (0.72–0.80)	0.72 (0.67–0.78)	0.793	67.3 (0.002)	<0.001	<0.001	0.54–1.07	No	Yes	Suggestive *
Gray 2016 (post-diagnostic)	Obs	11	21,030	0.84 (0.73–0.98)	0.85 (0.73–0.98)	0.84 (0.79–0.90)	0.90 (0.80–1.01)	0.760	69.0 (<0.001)	0.029	<0.001	0.53–1.34	No	No	Weak
Gray 2016 (pre-diagnostic)	Obs	6	44,026	0.85 (0.76–0.96)	0.85 (0.76–0.96)	0.84 (0.80–0.88)	0.85 (0.79–0.92)	0.720	76.0 (<0.001)	0.011	<0.001	0.59–1.24	No	Yes	Suggestive *
Ling 2015 (post-diagnostic)	Cohort	5	10,038	0.93 (0.68–1.27)	0.93 (0.68–1.27)	0.81 (0.73–0.90)	0.75 (0.66–0.84)	0.443	69.4 (0.011)	0.639	<0.001	0.33–2.59	No	No	Non-significant
Ling 2015 (pre-diagnostic)	Cohort	4	12,396	0.73 (0.61–0.88)	0.73 (0.62–0.86)	0.74 (0.66–0.84)	0.81 (0.68–0.96)	0.251	19.9 (0.291)	<0.001	<0.001	0.45–1.19	No	Yes	Suggestive
Cai 2015 (post & pre-diagnostic)	Obs	4	11,786	0.76 (0.61–0.95)	0.79 (0.65–0.95)	0.79 (0.65–0.95)	0.71 (0.54–0.94)	0.587	0.0 (0.447)	0.013	0.013	0.52–1.18	No	Yes	Suggestive
Cai 2015 (post-diagnostic)	Obs	4	15,862	0.76 (0.68–0.85)	0.76 (0.68–0.85)	0.76 (0.68–0.85)	0.75 (0.66–0.84)	0.723	0.0 (0.393)	<0.001	<0.001	0.60–0.96	No	Yes	Convincing
Cai 2015 (pre-diagnostic)	Obs	2	10,553	0.70 (0.54–0.91)	0.70 (0.54–0.91)	0.70 (0.54–0.91)	0.71 (0.54–0.94)	-	0.0 (0.795)	0.007	0.007	NA	-	Yes	-
Zhong 2015 (post-diagnostic)	Obs	6	12,441	0.96 (0.76–1.22)	0.97 (0.75–1.24)	0.86 (0.77–0.95)	0.75 (0.66–0.85)	0.295	65.9 (0.004)	0.792	0.003	0.45–2.09	No	No	Weak
Zhong 2015 (pre-diagnostic)	Obs	3	18,733	0.77 (0.66–0.89)	0.78 (0.69–0.88)	0.79 (0.72–0.87)	0.82 (0.74–0.91)	0.353	31.4 (0.300)	<0.001	<0.001	0.27–2.25	No	Yes	Suggestive
Endocrine-Related Gynecological Cancer															
Xie 2017	Obs	9	5449	-	0.70 (0.58–0.83)	0.71 (0.63–0.80)	0.66 (0.55–0.80)	0.250	33.3 (0.151)	<0.001	<0.001	0.47–1.04	No	Yes	Suggestive
Endometrial Cancer															
Xie 2017	Obs	4	3460	-	0.80 (0.62–1.03)	0.84 (0.69–1.01)	0.92 (0.70–1.20)	0.046	0.0 (0.680)	0.083	0.058	0.34–1.89	Yes	Yes	Non-significant
Zhong 2015 (post-diagnostic)	Obs	3	3261	0.89 (0.72–1.09)	0.86 (0.64–1.15)	0.89 (0.72–1.09)	0.92 (0.70–1.20)	0.156	0.0 (0.203)	0.309	0.255	0.05–13.49	No	Yes	Non-significant
Kidney Cancer															
Nayan 2017	Overall	7	11,491	0.74 (0.63–0.88)	0.74 (0.63–0.88)	0.78 (0.71–0.87)	0.80 (0.66–0.97)	0.057	51.8 (0.052)	0.001	<0.001	0.47–1.17	Yes	Yes	Weak
Luo 2015	Obs	3	5881	0.81 (0.68–0.96)	0.81 (0.69–0.96)	0.82 (0.72–0.94)	0.84 (0.69–1.00)	0.378	26.0 (0.260)	0.015	0.005	0.19–3.47	No	No	Suggestive
Lymphoma															
Zhong 2015 (post-diagnostic)	Obs	3	782	1.15 (0.85–1.55)	1.15 (0.85–1.55)	1.15 (0.85–1.55)	1.23 (0.88–1.71)	0.195	0.0 (0.602)	0.362	0.362	0.16–8.24	No	Yes	Non-significant
Ovarian Cancer															
Li 2018	Obs	7	16,389	0.74 (0.63–0.87)	0.74 (0.63–0.87)	0.79 (0.73–0.86)	0.81 (0.72–0.90)	0.061	55.0 (0.038)	<0.001	<0.001	0.49–1.11	No	Yes	Suggestive
Xie 2017	Obs	5	1989	-	0.63 (0.54–0.74)	0.63 (0.54–0.74)	0.66 (0.55–0.80)	0.200	0.0 (0.680)	<0.001	<0.001	0.48–0.82	No	Yes	Convincing
Zhong 2015 (post-diagnostic)	Obs	2	276	0.39 (0.22–0.71)	0.39 (0.22–0.71)	0.39 (0.22–0.71)	0.24 (0.07–0.87)	-	0.0 (0.395)	0.002	0.002	NA	-	Yes	-
Pancreatic Cancer															
Jian-Yu 2018	Overall	6	12,057	0.75 (0.59–0.90)	0.81 (0.69–0.95)	0.92 (0.87–0.97)	0.94 (0.89–1.01)	0.008	81.1 (<0.001)	0.009	0.001	0.52–1.26	Yes	No	Weak
Prostate Cancer															
Mei 2017	Cohort	10	73,716	0.72 (0.63–0.81)	0.72 (0.63–0.81)	0.89 (0.87–0.91)	0.79 (0.76–0.82)	0.044	95.0 (<0.001)	<0.001	<0.001	0.46–1.12	Yes	Yes	Weak
Meng 2016 (post-diagnostic)	Obs	7	58,838	0.84 (0.71–0.99)	0.77 (0.68–0.86)	0.80 (0.76–0.83)	0.79 (0.75–0.83)	0.376	72.0 (0.002)	<0.001	<0.001	0.54–1.09	No	Yes	Suggestive *
Meng 2016 (pre-diagnostic)	Obs	2	1337	0.56 (0.38–0.85)	0.56 (0.38–0.83)	0.56 (0.38–0.83)	0.57 (0.38–0.85)	-	0.0 (0.770)	0.004	0.004	NA	-	Yes	-
Raval 2016	Cohort	6	31,539	0.76 (0.63–0.90)	0.76 (0.63–0.91)	0.80 (−0.75–0.86)	0.86 (0.78–0.95)	0.503	71.0 (0.004)	0.004	<0.001	0.44–1.29	No	Yes	Weak
Luo 2015	Obs	5	22,439	0.82 (0.70–0.97)	0.83 (0.70–0.97)	0.85 (0.78–0.93)	0.86 (0.78–0.95)	0.461	46.0 (0.110)	0.022	<0.001	0.52–1.31	No	Yes	Suggestive
Zhong 2015 (post-diagnostic)	Obs	3	18,814	0.59 (0.35–0.99)	0.59 (0.34–1.01)	0.82 (0.75–0.90)	0.86 (0.78–0.95)	0.228	84.1 (0.001)	0.053	<0.001	0.00–406.64	No	No	Non-significant
Urothelial Tract Cancer															
Zhong 2015 (post-diagnostic)	Obs	5	9488	0.87 (0.75–1.00)	0.87 (0.75–1.00)	0.87 (0.79–0.95)	0.89 (0.71–1.12)	0.917	52.8 (0.070)	0.049	0.001	0.56–1.34	No	No	Weak

ES, Effect size; CI, Confidence interval; PI, Prediction interval; Obs, Observational study. § Risk ratio (95% Confidence interval) of the largest study in each meta-analysis. † I^2^ metric of inconsistency (95% confidence interval of I^2^) and *P*-value of the Cochran Q test for evaluation of heterogeneity. * Convincing or suggestive level of evidence due to the greater number of studies that decrease risk.

**Table 2:** Summary of each individual meta-analysis on associations of the use of statin and cancer-specific mortality in various cancers.

Type/Author, Year	Study Design	No of Study	No of Total Participants	Random Effects (Reported) (ES, 95%CI)	Random Effects (Re-Analyzed) (ES, 95%CI)	Fixed Effects (Re-Analyzed) (ES, 95%CI)	Largest Effect §	Egger	I^2^ (*P*) †	*P* (Random)	*P* (Fixed)	95% PI (Random)	Small Study Effect	Same Direction	Evidence
Bladder cancer															
Luo 2015	Obs	2	2619	1.06 (0.87–1.29)	1.06 (0.87–1.29)	1.06 (0.87–1.29)	1.04 (0.84–1.28)	-	0.0 (0.590)	0.559	0.559	NA	-	Yes	Non-significant
Breast cancer															
Liu 2017	Cohort	8	196,120	0.73 (0.59–0.92)	0.73 (0.58–0.92)	0.73 (0.67–0.78)	0.85 (0.74–0.98)	0.997	85.6 (<0.001)	0.007	<0.001	0.34–1.58	No	Yes	Weak
Manthravadi 2016	Cohort	6	46,970	0.30 (0.46–1.06)	0.69 (0.45–1.06)	0.62 (0.54–0.71)	0.35 (0.28–0.45)	0.591	86.0 (<0.001)	0.091	<0.001	0.16–2.92	No	No	Non-significant
Mansourian 2016	Obs	13	99,610	0.85 (0.83–0.87)	0.85 (0.82–0.88)	0.85 (0.83–0.87)	0.83 (0.80–0.86)	0.465	8.6 (0.360)	<0.001	<0.001	NA	No	Yes	-
Zhong 2015 (post-diagnostic)	Obs	3	49,116	0.60 (0.41–0.88)	0.60 (0.39–0.92)	0.60 (0.52–0.69)	0.47 (0.39–0.57)	0.995	84.1 (<0.001)	0.018	<0.001	0.00–106.05	No	Yes	Weak
Zhong 2015 (pre-diagnostic)	Obs	4	88,235	0.73 (0.61–0.89)	0.77 (0.68–0.87)	0.77 (0.68–0.87)	0.60 (0.35–1.01)	0.002	21.5 (0.428)	<0.001	<0.001	0.59–1.01	Yes	No	Suggestive
Colorectal cancer															
Gray 2016 (post-diagnostic)	Obs	4	19,152	0.84 (0.68–1.04)	0.84 (0.68–1.04)	0.82 (0.75–0.91)	0.90 (0.77–1.05)	0.887	67.0 (0.030)	0.118	<0.001	0.36–2.00	No	Yes	Non-significant
Gray 2016 (pre-diagnostic)	Obs	6	86,622	0.82 (0.79–0.86)	0.82 (0.79–0.86)	0.82 (0.79–0.86)	0.81 (0.75–0.88)	0.152	0.0 (0.570)	<0.001	<0.001	NA	No	Yes	-
Ling 2015 (post-diagnostic)	Cohort	3	8667	0.70 (0.60–0.81)	0.70 (0.60–0.82)	0.70 (0.60–0.82)	0.71 (0.61–0.84)	0.219	0.0 (0.535)	<0.001	<0.001	0.26–1.87	No	Yes	Suggestive
Ling 2015 (pre-diagnostic)	Cohort	6	74,042	0.80 (0.77–0.84)	0.80 (0.77–0.84)	0.80 (0.77–0.84)	0.79 (0.74–0.85)	0.231	10.8 (0.347)	<0.001	<0.001	0.74–0.88	No	Yes	Convincing
Cai 2015 (pre&post-diagnostic)	Obs	6	69,949	0.80 (0.75–0.85)	0.80 (0.75–0.85)	0.80 (0.77–0.85)	0.79 (0.74–0.85)	0.172	19.3 (0.288)	<0.001	<0.001	0.71–0.90	No	Yes	Convincing
Cai 2015 (post-diagnostic)	Obs	3	15,023	0.70 (0.60–0.81)	0.70 (0.60–0.82)	0.70 (0.60–0.82)	0.71 (0.61–0.84)	0.219	0.0 (0.535)	<0.001	<0.001	0.26–1.87	No	Yes	Suggestive
Cai 2015 (pre-diagnostic)	Obs	5	69,375	0.80 (0.74–0.86)	0.80 (0.74–0.86)	0.81 (0.77–0.85)	0.79 (0.74–0.85)	0.298	28.3 (0.233)	<0.001	<0.001	0.67–0.95	No	Yes	Convincing
Zhong 2015 (post-diagnostic)	Obs	4	11,070	0.79 (0.58–1.08)	0.79 (0.58–1.08)	0.77 (0.67–0.88)	0.71 (0.61–0.83)	0.959	60.5 (0.058)	0.141	<0.001	0.24–2.65	No	No	Weak
Zhong 2015 (pre-diagnostic)	Obs	3	25,081	0.82 (0.73–0.91)	0.82 (0.74–0.90)	0.83 (0.78–0.89)	0.77 (0.68–0.88)	0.414	36.2 (0.239)	<0.001	<0.001	0.31–2.19	No	Yes	Suggestive
Endocrine gynecological cancer															
Xie 2017	Obs	4	1079	-	0.75 (0.55–1.01)	0.72 (0.58–0.90)	0.74 (0.54–1.02)	0.357	35.1 (0.202)	0.057	0.004	0.27–2.09	No	Yes	Non-significant
Kidney cancer															
Nayan 2017	Overall	6	10,337	0.67 (0.47–0.94)	0.67 (0.48–0.94)	0.81 (0.71–0.93)	0.85 (0.72–1.01)	0.120	67.0 (0.010)	0.022	0.003	0.25–1.82	No	No	Weak
Luo 2015	Obs	2	3273	0.71 (0.35–1.50)	0.72 (0.35–1.51)	0.84 (0.64–1.11)	1.02 (0.74–1.39)	–	82.0 (0.020)	0.389	0.222	NA	-	Yes	Non-significant
Ovarian cancer															
Li 2018	Obs	3	27,690	0.87 (0.80–0.95)	0.87 (0.80–0.95)	0.87 (0.80–0.95)	0.93 (0.81–1.08)	0.577	0.0 (0.411)	0.002	0.002	0.50–1.54	No	No	Weak
Prostate cancer															
Meng 2016 (post-diagnostic)	Obs	4	57,058	0.64 (0.52–0.79)	0.64 (0.52–0.79)	0.73 (0.69–0.77)	0.74 (0.70–0.79)	0.254	82.0 (<0.001)	<0.001	<0.001	0.27–1.55	No	Yes	Suggestive *
Meng 2016 (pre-diagnostic)	Obs	6	35,684	0.53 (0.29–0.98)	0.54 (0.37–0.78)	0.78 (0.72–0.84)	0.81 (0.75–0.88)	0.019	77.0 (<0.001)	0.001	<0.001	0.18–1.64	Yes	Yes	Weak
Raval 2016	Cohort	5	21,306	0.76 (0.64–0.89)	0.76 (0.64–0.89)	0.76 (0.69–0.84)	0.76 (0.66–0.88)	0.593	30.0 (0.150)	0.001	<0.001	0.49–1.17	No	Yes	Suggestive
Luo 2015	Obs	7	28,897	0.70 (0.59–0.83)	0.70 (0.60–0.83)	0.74 (0.68–0.82)	0.76 (0.66–0.88)	0.011	43.0 (0.100)	<0.001	<0.001	0.48–1.04	Yes	Yes	Suggestive
Zhong 2015 (post-diagnostic)	Obs	3	19,322	0.77 (0.70–0.85)	0.77 (0.70–0.85)	0.77 (0.70–0.85)	0.76 (0.66–0.88)	0.973	0.0 (0.970)	<0.001	<0.001	0.38–1.54	No	Yes	Suggestive
Zhong 2015 (pre-diagnostic)	Obs	3	5460	0.44 (0.20–0.93)	0.44 (0.21–0.92)	0.72 (0.62–0.82)	0.78 (0.67–0.90)	0.148	86.3 (0.001)	0.029	<0.001	Not estimable	No	Yes	Suggestive *
Urothelial tract cancer															
Zhong 2015 (post-diagnostic)	Obs	4	6880	0.86 (0.65–1.16)	0.87 (0.66–1.14)	0.87 (0.76–1.01)	0.86 (0.72–1.03)	0.901	61.8 (0.073)	0.307	0.070	0.30–2.53	No	Yes	Non-significant

ES, Effect size; CI, Confidence interval; PI, Prediction interval; Obs, Observational study. § Risk ratio (95% Confidence interval) of the largest study in each meta-analysis. † I^2^ metric of inconsistency (95% confidence interval of I^2^) and *P*-value of the Cochran Q test for evaluation of heterogeneity. * Convincing or suggestive level of evidence due to the greater number of studies that decrease risk.

**Table 3:** Summary of each individual meta-analysis on associations of the use of statin and recurrence-free survival, progression-free survival and disease-free survival in various cancers.

Type/Author, Year	Study Design	No of Study	No of Total Participants	Random Effects (Reported) (ES, 95%CI)	Random Effects (Re-Analyzed) (ES, 95%CI)	Fixed Effects (Re-Analyzed) (ES, 95%CI)	Largest Effect §	Egger	I^2^ (*P*) †	*P* (Random)	*P* (Fixed)	95% PI (Random)	Small Study Effect	Same Direction	Evidence
**Recurrence-free survival**															
Bladder cancer															
Luo 2015	Obs	3	3571	1.05 (0.94–1.18)	1.06 (0.94–1.19)	1.06 (0.94–1.19)	1.04 (0.96–1.24)	0.844	0.0 (0.950)	0.375	0.375	0.47–2.36	No	Yes	Non-significant
Breast cancer															
Manthravadi 2016	Cohort	10	32,373	0.64 (0.53–0.79)	0.64 (0.52–0.79)	0.69 (0.60–0.79)	0.80 (0.64–1.00)	0.093	44.0 (0.070)	<0.001	<0.001	0.38–1.09	Yes	Yes	Suggestive
Colorectal cancer															
Cai 2015	Obs	2	1233	0.98 (0.36–2.70)	0.98 (0.36–2.70)	1.12 (0.58–2.15)	1.28 (0.64–2.54)	–	26.1 (0.345)	0.975	0.730	NA	-	Yes	Non-significant
Kidney cancer															
Nayan 2017	Overall	4	2197	0.97 (0.89–1.06)	0.97 (0.89–1.06)	1.00 (0.99–1.01)	1.09 (0.65–1.81)	0.364	55.2 (0.082)	0.524	0.899	0.70–1.36	No	Yes	Non-significant
Luo 2015	Obs	3	5080	0.91 (0.54–1.55)	0.91 (0.54–1.55)	1.00 (0.81–1.23)	1.22 (0.95–1.57)	0.783	72.0 (0.030)	0.736	0.991	0.47–2.36	No	Yes	Non-significant
Prostate cancer															
Park 2013	Cohort	13	21,185	0.90 (0.74–1.08)	0.90 (0.74–1.08)	0.92 (0.84–1.00)	0.99 (0.83–1.18)	0.649	69.6 (<0.001)	0.252	0.057	0.48–1.67	No	Yes	Non-significant
**Progression-free survival**															
Bladder cancer															
Luo 2015	Obs	2	2069	0.87 (0.65–1.15)	0.87 (0.65–1.15)	0.87 (0.65–1.15)	0.77 (0.52–1.13)	0.461	0.0 (0.370)	0.320	0.320	NA	No	Yes	Non-significant
Endocrine gynecological cancer															
Xie 2017	Obs	3	421	-	0.69 (0.46–1.02)	0.68 (0.49–0.93)	0.65 (0.39–1.07)	0.439	33.6 (0.222)	0.066	0.018	0.02–27.87	No	Yes	Non-significant
Kidney cancer															
Nayan 2017	Overall	2	4965	0.92 (0.51–1.65)	0.92 (0.51–1.65)	1.00 (0.82–1.23)	0.67 (0.47–0.96)	–	86.2 (0.007)	0.772	0.996	NA	-	No	Non-significant
Prostate cancer															
Luo 2015	Obs	5	6032	0.84 (0.62–1.14)	0.84 (0.62–1.14)	0.87 (0.71–1.05)	1.10 (0.78–1.56)	0.607	52 (0.080)	0.260	0.148	0.34–2.10	No	Yes	Non-significant
**Disease-free survival**															
Colorectal cancer														
Cai 2015	Obs	2	1233	1.13 (0.78–1.62)	1.13 (0.78–1.62)	1.13 (0.78–1.62)	1.07 (0.68–1.67)	-	0.0 (0.691)	0.514	0.514	NA	-	Yes	Non-significant

ES, Effect size; CI, Confidence interval; PI, Prediction interval; Obs, Observational study; § Risk ratio (95% Confidence interval) of the largest study in each meta-analysis. † I^2^ metric of inconsistency (95% confidence interval of I^2^) and *P*-value of the Cochran Q test for evaluation of heterogeneity.

**Table 4:** Summary of the meta-analysis results by pooling all the datasets on associations of statin and the mortality or survival.

Cancer Type	No of Studies	No of Total Participants	Random Effects (RR, 95%CI)	*P* (Random)	Fixed Effects (RR, 95%CI)	*P* (Fixed)	Largest Effect § (RR, 95%CI)	D/N/I	Egger	I^2^ (*P*) †	95% PI (Random)	95% PI (Fixed)	Concordant Direction	Evidence
**All-cause mortality**														
Bladder cancer	1	1117	1.14 (0.89–1.44)	0.286	1.14 (0.89–1.44)	0.286	1.14 (0.89–1.44)	0/1/0	-	-	NA	NA	No	Non-significant
Breast cancer	20	160,806	0.65 (0.55–0.77)	<0.001	0.66 (0.62–0.70)	<0.001	0.54 (0.44–0.67)	12/0/8	0.787	85.2 (<0.001)	0.33–1.29	0.34–1.28	Yes	Suggestive *
Colorectal cancer	24	85,231	0.81 (0.75–0.88)	<0.001	0.82 (0.80–0.86)	<0.001	0.82 (0.74–0.90)	15/8/1	0.444	68.1 (<0.001)	0.60–1.10	0.61–1.11	Yes	Suggestive *
Endocrine-related gynecological cancer	9	5449	0.70 (0.58–0.83)	<0.001	0.71 (0.63–0.80)	<0.001	0.66 (0.55–0.80)	4/5/0	0.250	33.3 (0.151)	0.47–1.04	0.49–1.03	Yes	Suggestive
Endometrial cancer	4	3460	0.80 (0.62–1.03)	0.083	0.84 (0.69–1.01)	0.058	0.92 (0.70–1.20)	1/3/0	0.046	36.1 (0.196)	0.34–1.89	0.38–1.82	Yes	Non-significant
Kidney cancer	7	11,491	0.74 (0.63–0.88)	0.001	0.78 (0.71–0.87)	<0.001	0.80 (0.66–0.98)	4/3/0	0.057	51.8 (0.053)	0.47–1.17	0.51–1.20	Yes	Weak
Lymphoma	3	782	1.15 (0.85–1.55)	0.362	1.15 (0.85–1.55)	0.362	1.23 (0.88–1.71)	0/3/0	0.195	0.0 (0.602)	0.16–8.24	0.16–8.24	Yes	Non-significant
Ovarian cancer	7	16,307	0.74 (0.63–0.87)	<0.001	0.79 (0.73–0.86)	<0.001	0.81 (0.72–0.90)	4/3/0	0.067	0.0 (0.411)	0.49–1.12	0.55–1.15	Yes	Suggestive
Pancreatic cancer	6	12,057	0.81 (0.69–0.95)	0.009	0.92 (0.87–0.97)	0.001	0.94 (0.89–1.01)	3/3/0	0.008	81.1 (<0.001)	0.52–1.26	0.62–1.36	No	Weak
Prostate cancer	21	95,128	0.73 (0.67–0.81)	<0.001	0.89 (0.88–0.91)	<0.001	0.79 (0.75–0.83)	15/6/0	0.002	89.9 (<0.001)	0.50–1.08	0.61–1.30	No	Weak
Urothelial tract cancer	5	9488	0.87 (0.75–1.00)	0.049	0.87 (0.79–0.95)	0.001	0.89 (0.71–1.12)	2/3/0	0.917	52.8 (0.070)	0.56–1.34	0.58–1.29	No	Weak
**Cancer-specific mortality**														
Bladder cancer	2	2619	1.06 (0.87–1.29)	0.559	1.06 (0.87–1.29)	0.559	1.04 (0.84–1.28)	0/2/0	-	0.0 (0.590)	NA	NA	Yes	Non-significant
Breast cancer	28	424,694	0.71 (0.65–0.78)	<0.001	0.82 (0.80–0.84)	<0.001	0.83 (0.80–0.86)	12/16/0	0.044	84.0 (<0.001)	0.50–1.02	0.58–1.16	Yes	Weak
Colorectal cancer	13	118,996	0.81 (0.78–0.85)	<0.001	0.82 (0.79–0.85)	<0.001	0.77 (0.69–0.87)	8/5/0	0.282	26.2 (0.180)	0.72–0.92	0.74–0.90	Yes	Convincing
Endocrine-related gynecological cancer	4	1079	0.75 (0.55–1.01)	0.057	0.72 (0.58–0.90)	0.004	0.74 (0.54–1.02)	1/3/0	0.357	35.1 (0.202)	0.27–2.09	0.29–1.82	Yes	Non-significant
Kidney cancer	6	10,337	0.67 (0.48–0.94)	0.022	0.81 (0.71–0.93)	0.003	0.85 (0.72–1.01)	3/3/0	0.120	66.6 (0.011)	0.25–1.82	0.33–1.98	No	Weak
Ovarian cancer	3	27,690	0.87 (0.80–0.95)	0.002	0.87 (0.80–0.95)	0.002	0.93 (0.81–1.08)	1/2/0	0.577	0.0 (0.411)	0.50–1.54	0.50–1.54	No	Weak
Prostate cancer	15	101,378	0.66 (0.58–0.74)	<0.001	0.74 (0.71–0.78)	<0.001	0.74 (0.70–0.79)	11/4/0	0.010	68.3 (<0.001)	0.47–0.93	0.54–1.02	Yes	Weak
Urothelial tract cancer	4	6880	0.87 (0.66–1.14)	0.307	0.87 (0.76–1.01)	0.070	0.86 (0.72–1.03)	1/3/0	0.901	61.8 (0.073)	0.30–2.53	0.34–2.22	Yes	Non-significant
**Recurrence-free survival**														
Bladder cancer	3	3571	1.06 (0.94–1.19)	0.375	1.06 (0.94–1.19)	0.375	1.04 (0.96–1.24)	0/3/0	0.844	0.0 (0.950)	0.47–2.36	0.47–2.36	Yes	Non-significant
Breast cancer	10	32,373	0.64 (0.52–0.79)	<0.001	0.69 (0.60–0.79)	<0.001	0.80 (0.64–1.00)	6/4/0	0.093	44.0 (0.070)	0.38–1.09	0.42–1.14	Yes	Weak
Colorectal cancer	2	1233	0.98 (0.36–2.70)	0.975	1.12 (0.58–2.15)	0.730	1.28 (0.64–2.54)	0/2/0	-	26.1 (0.345)	NA	NA	Yes	Non-significant
Kidney cancer	4	2197	0.97 (0.89–1.06)	0.524	1.00 (0.99–1.01)	0.899	1.09 (0.65–1.81)	1/3/0	0.364	56.8 (0.074)	0.70–1.36	0.36–1.31	Yes	Non-significant
Prostate cancer	13	21,185	0.90 (0.74–1.08)	0.252	0.92 (0.84–1.00)	0.057	0.99 (0.83–1.18)	5/7/1	0.649	69.6 (<0.001)	0.48–1.67	0.50–1.66	Yes	Non-significant
**Progression-free survival**														
Bladder cancer	2	2069	0.87 (0.65–1.15)	0.320	0.87 (0.65–1.15)	0.320	0.77 (0.52–1.13)	0/2/0	0.461	0.0 (0.370)	NA	NA	Yes	Non-significant
Endocrine-related gynecological cancer	3	421	0.69 (0.46–1.02)	0.066	0.68 (0.49–0.93)	0.018	0.65 (0.39–1.07)	1/2/0	0.439	33.6 (0.222)	0.02–27.87	0.02–19.47	Yes	Non-significant
Kidney cancer	2	4965	0.92 (0.51–1.65)	0.772	1.00 (0.82–1.23)	0.996	0.67 (0.47–0.96)	1/1/0	-	86.2 (0.007)	NA	NA	No	Non-significant
Prostate cancer	5	6032	0.84 (0.62–1.14)	0.260	0.87 (0.71–1.05)	0.148	1.10 (0.78–1.56)	2/3/0	0.607	52 (0.080)	0.34–2.10	0.38–2.00	Yes	Non-significant
**Disease-free survival**														
Colorectal cancer	2	1,233	1.13 (0.78–1.62)	0.514	1.13 (0.78–1.62)	0.514	1.07 (0.68–1.67)	0/2/0	-	0.0 (0.691)	NA	NA	Yes	Non-significant

D/N/I, Decreasing risk/No difference/Increasing risk; RR, Risk ratio; CI, Confidence interval; PI, Prediction. § Risk ratio (95% Confidence interval) of the largest study in each meta-analysis. † I^2^ metric of inconsistency (95% confidence interval of I^2^) and *P*-value of the Cochran Q test for evaluation of heterogeneity. * Suggestive or convincing level of evidence due to the greater number of studies that decrease risk in which a high heterogeneity is due to differences in the effect size of the association.

**Table 5:** Evidence of association between statin use and mortality or survival outcomes.

Evidence Category	All-Cause Mortality *	Cancer-Specific Mortality	Recurrence Free Survival	Progression-Free Survival	Disease-Free Survival
Convincing	-	Colorectal cancer (0.82; 0.79–0.85)	-	-	-
Suggestive	Breast cancer (0.65; 0.55–0.77) Colorectal cancer (0.82; 0.75–0.88) Endocrine-related gynecological cancer (0.71; 0.58–0.83) Ovarian cancer (0.74; 0.63–0.87)	-	-	-	-
Weak	Kidney cancer (0.73; 0.71–0.87) Pancreatic cancer (0.81; 0.69–0.95) Prostate cancer (0.89; 0.88–0.91) Urothelial tract cancer (0.87; 0.75–1.00)	Breast cancer (0.71; 0.65–0.78) Kidney cancer (0.67; 0.48–0.94) Ovarian cancer (0.87; 0.80–0.95) Prostate cancer (0.66; 0.58–0.74)	Breast cancer (0.64; 0.52–0.79)	-	-
Non-significant	Bladder cancer Endometrial cancer Lymphoma	Bladder cancer Endocrine-related gynecological cancer Urothelial tract cancer	Bladder cancer Colorectal cancer Kidney cancer Prostate cancer	Bladder cancer Endocrine-related gynecological cancer Kidney cancer Prostate cancer	Colorectal cancer

* Results with statistically significant association (convincing, suggestive and weak) were presented with its random summary effects and 95% confidence interval.

## Supplementary Materials

The supplementary material is available online at https://www.mdpi.com/2077-0383/9/2/326/s1.
